# Editorial: Redefining acute psychiatric care: strategies for improved inpatient experiences, vol II

**DOI:** 10.3389/fpsyt.2026.2032873

**Published:** 2026-10-27

**Authors:** Alfonso Ceccherini-Nelli, Stefano Barlati

**Affiliations:** 1 Alberta Hospital Edmonton, University of Alberta, Edmonton, AB, Canada; 2 Department of Clinical and Experimental Sciences, University of Brescia, Brescia, Italy; 3 Department of Mental Health and Addiction Services, ASST Spedali Civili of Brescia, Brescia, Italy

**Keywords:** acute psychiatric care, coercion, inpatient experience, involuntary treatment, readmission, stigma, substance use disorder, ward environment

Any effort to redefine acute care must start with the capacity to deliver it. In a two-round Delphi survey of senior clinicians in Hong Kong’s public system, Ma et al. reached consensus on an optimal 68 adult psychiatric beds per 100,000 population, 39% above current provision, which sits near the agreed minimum of 50, plus a daytime nurse-to-patient ratio of 1:4 and target stays of around three weeks for psychotic and mood disorders. Bed need was judged to depend chiefly on population needs, not on community services alone.

Patients with medical comorbidity, one of the Research Topic’s at-risk groups, are vulnerable from the first contact. Mesbah et al. reviewed 507 Houston emergency department encounters with a purely behavioural presenting complaint. A global statement such as “neurologically intact” appeared in 94.5% of charts. Yet the Glasgow Coma Scale was recorded in only 9.3%, deep tendon reflexes in 1.4%, and cognition and memory in 7.5%. Nothing had improved since a 2016 audit, suggesting diagnostic overshadowing and inequity.

Substance use disorder, another at-risk condition, is closely tied to compulsory care. Among 5,887 admissions to a Turkish referral centre over 2019-2023, Akdag et al. found substance use in 27.5%, mostly polysubstance. Involuntary admissions rose from 15% in 2019 to 36–40% in 2022-2023. Substance use disorder (OR 3.6), substance-induced psychosis (OR 2.3), male sex and calendar year independently predicted involuntary admission, while bipolar disorder predicted lower risk. Use of atypical and depot antipsychotics, clozapine and ECT grew, yet patients with substance use disorders were less likely to receive clozapine, mood stabilisers or ECT.

Preventing hetero-aggression depends on recognising who is at risk. Liu et al. studied 555 men hospitalised for alcohol dependence in Hefei, China. More than a third (37.1%) had at least one documented aggressive incident, and a third of these had three or more. After adjustment for withdrawal severity, three factors were independently associated with aggression: shorter sleep, higher nurse-rated suicide risk, and lower haemoglobin, even within the normal range. Explained variance was modest, but these markers are cheap, routine and potentially modifiable.

Cossu et al. address patients’ legal rights and the ethics of coercion directly, in the context of Article 12 of the UN Convention on the Rights of Persons with Disabilities. Rather than asking whether involuntary treatment is ever justified, they propose treating each instance as a sentinel event. Each would be reported, examined by root cause analysis with service users and families, and followed by an action plan. The model locates the drivers of coercion in resources, training and power asymmetries, not solely in the patient’s symptoms.

The ward environment is one such driver, and it bears directly on restraint. Liu et al. used a natural experiment: renovations closed the outdoor areas of two New York inpatient units for several months in 2024. On the general adult unit, the loss of outdoor access coincided with more intramuscular PRN orders, seclusion or restraint, and constant observation. On the geriatric unit, only oral PRN use increased. Although confounding cannot be excluded, outdoor space appears to be a de-escalation resource, not an amenity.

Amann et al. extend this to therapy, reviewing nature-based interventions such as forest walks, gardening and outdoor activity on psychiatric wards. The five inpatient studies are mostly small, uncontrolled and focused on depression; they show short-term mood gains and suggest meaning, agency, belonging and calm as mechanisms. Adverse events were never systematically recorded, so powered randomised trials with safety monitoring are needed.

Whether the hospital is a place where patients feel well cared for is a question only patients can answer. Cuevas-Esteban et al. applied Patient Journey Mapping to a 14-bed unit near Barcelona that uses Safewards and opens its doors six hours a day; 60% of participants were involuntarily admitted. Focus groups (n = 18) and ratings (n = 32) identified emergency waiting and group activities as “moments of pain”. Medical visits, door openings and discharge were high points. Patients objected less to the duration of waiting than to insufficient/inconsistent information and public, communal nature of medication administration routines, both remediable at low cost.

Treating admission as one phase of a continuing care project means planning for what follows it. In a Málaga case-control study of 196 episodes, García-Illanes et al. combined seven variables into a points-based index for 30-day readmission: previous admissions, personality disorder, social risk, emergency attendance in the prior year, legal capacity restriction, aggression towards others at admission, and being married or widowed. The index discriminated moderately well (AUC 0.76; optimism-corrected 0.73). Its three risk levels can guide the intensity of post-discharge follow-up, pending external validation.

Finally, the Research Topic’s emphasis on staff training reaches back to undergraduate education. Wang et al.’s integrative review of 25 studies found moderate-to-high mental health stigma among nursing students, with wide cross-cultural variation. Education, simulation, digital methods, peer support and clinical placements all helped. Contact benefits could fade or reverse after distressing first exposures; multicomponent programmes combining contact with guided reflection and supervision appear most promising.

Read together, these contributions suggest that the acute ward is shaped as much by what surrounds the patient as by what is prescribed. Coercion is the collection’s through-line: it rises with substance use and male sex, can be anticipated by simple bedside markers, increases when outdoor space is lost, and is softened, though not abolished, by open doors and clear information. Yet redefining acute care also requires the capacity to deliver it: Ma et al. remind us that shorter stays and community alternatives cannot substitute for adequate beds and staffing. Three gaps remain. First, most studies are single-centre and observational, and few test interventions prospectively. Second, the outcomes this Research Topic set out to capture, psychosocial functioning and personal recovery after discharge, are still rarely measured. Third, the perspectives of patients, families and staff need to be brought into the same study designs. Overall, acute psychiatric care should be judged not only by its safety and efficiency, but also by its respect for autonomy and dignity and the quality of the inpatient experience.

